# The potential roles of cigarette smoke-induced extracellular vesicles in oral leukoplakia

**DOI:** 10.1186/s40001-023-01217-0

**Published:** 2023-07-22

**Authors:** Qiao Peng, Ning Duan, Xiang Wang, Wenmei Wang

**Affiliations:** Department of Oral Medicine, Nanjing Stomatological Hospital, Medical School of Nanjing University, 30 Zhongyang Road, Nanjing, 210008 China

**Keywords:** Oral leukoplakia, Cigarette smoke, Extracellular vesicles, Malignant transformation

## Abstract

**Background:**

The onset of oral leukoplakia (OLK), the most common oral lesion with a high risk of malignant transformation, is closely associated with the exposure of cigarette smoke. Cigarette smoke is a complicated mixture of more than 4500 different chemicals including various oxidants and free radical, which contributes to the onset of immune and inflammatory response or even carcinogenesis. Recent studies have proved that the exposure of cigarette smoke leads to the onset and aggravation of many diseases via significantly changed the production and components of extracellular vesicles. The extracellular vesicles are membrane-enclosed nanosized particles secreted by diverse cells and involved in cell–cell communication because of their ability to deliver a number of bioactive molecules including proteins, lipids, DNAs and RNAs. Getting insight into the mechanisms of extracellular vesicles in regulating OLK upon cigarette smoke stimulation contributes to unravel the pathophysiology of OLK in-depth. However, evidence done on the role of extracellular vesicles in cigarette smoke-induced OLK is still in its infancy.

**Materials and methods:**

Relevant literatures on cigarette smoke, oral leukoplakia and extracellular vesicles were searched in PubMed database.

**Conclusions:**

In this review, we summarize the recent findings about the function of extracellular vesicles in the pathogenesis of cigarette smoke-induced diseases, and to infer their potential utilizations as diagnostic biomarkers, prognostic evaluation, and therapeutic targets of OLK in the future.

## Introduction

A robust body of evidence have accumulated documenting that cigarette smoke (CS) is an essential risk factor for multiple human diseases worldwide including cancers, cardiovascular disease, chronic lung diseases, asthma and inflammatory skin disorders [1–4]. The prevalent use of CS and the series of diseases induced by CS result in serious economic and health burden to the world, especially for the undeveloped countries [5]. A cross-over study performed by Kerr et al. found that the heart rate of healthy individuals increased only after 4 h use of tobacco smoking/electronic cigarettes [6]. Moreover, the peak expiratory flow remarkably down-regulated in electronic cigarettes group [6]. These data suggested the acute exposure to CS influenced vascular and respiratory function. It is reported that CS is a complicated mixture of chemicals that contains about 10^15–17^ oxidants/free radicals and up to 4500–4700 different compounds, such as nicotine, tobacco-specific nitrosamines, reactive aldehydes and quinones, per puff [7, 8]. Most of these components manifest great cellular toxicity pathogenesis by modulating the cellular survival, apoptosis, ferroptosis, autophagy, proliferation and angiogenesis [9–11]. They also act as mutagen and carcinogen factors promoting tumorigenesis [12, 13].

The tissues of oral cavity are always inevitably to suffer damage, because it is the first part stimulated by CS. In fact, CS is one of the most established major risk factors for many oral diseases, including periodontal disease, dental caries, aphthous ulcers, primary Sjögren’s syndrome and peri-implantitis [14–16]. According to a meta-analysis performed by Leite et al., smoking increased the risk of periodontitis by 85% and this risk could be decreased by about 14% if smoking factor is eliminated in this population, which well-demonstrated the detrimental effect of smoking on periodontitis [17]. CS also represents the most important etiological factor that promotes oral potentially malignant disorders (OPMD) and oral squamous cell carcinoma (OSCC) [12, 18, 19]. Among the OPMDs, oral leukoplakia (OLK) is the most common potentially premalignant lesion that showing a primary white change of oral mucosa [20, 21]. The prevalence for OLK is between 1.5% and 2.6%, and the risk malignant transformation is up to 5–36% [22, 23]. Strong evidence have been found that the incidence of OLK was closely associated with the addiction of tobacco [24, 25]. In addition, the correlation between CS and OLK is underlined by a dose-dependent manner [26]. The complicated pathogenic impacts of CS are considered as the attribution that leads to OLK and even its malignant transformation, In a prior study by Ye et al., they found that the immortalized human oral mucosa epithelial cell line leuk1 could significantly accumulated reactive oxygen species (ROS) upon cigarette smoke extract (CSE) exposure compared to the unexposed co-cultured cells, indicating the destructive effect of oxidative stress on the structure and function of oral epithelium [27].

**Fig. 1:**
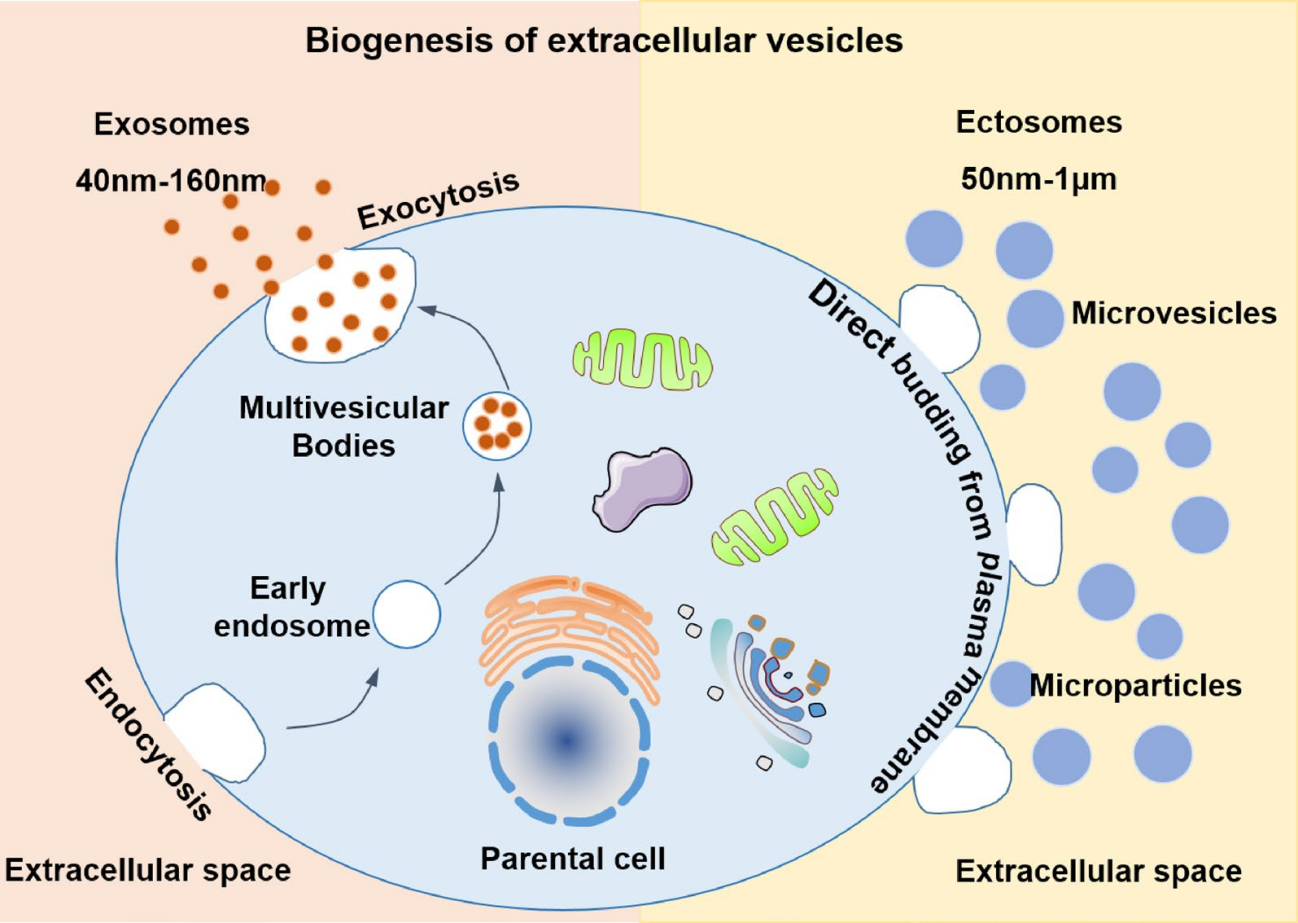
Biogenesis of extracellular vesicles. Extracelluar vesicles (EVs) are a heterogeneous group of cell-derived membranous structures that are categories as exosomes and ectosomes according to their different mechanism of formation and approximate size. Exosomes are EVs in the size range of 40–160 nm in diameter with an endosomal origin and generate by the fusion of multivesicular bodies, whereas ectosomes, including microvesicles and microparticles, are ubiquitous vesicles directly released from the plasma membrane through outward budding with a size range of 50 nm–1 μm in diameter

**Fig. 2:**
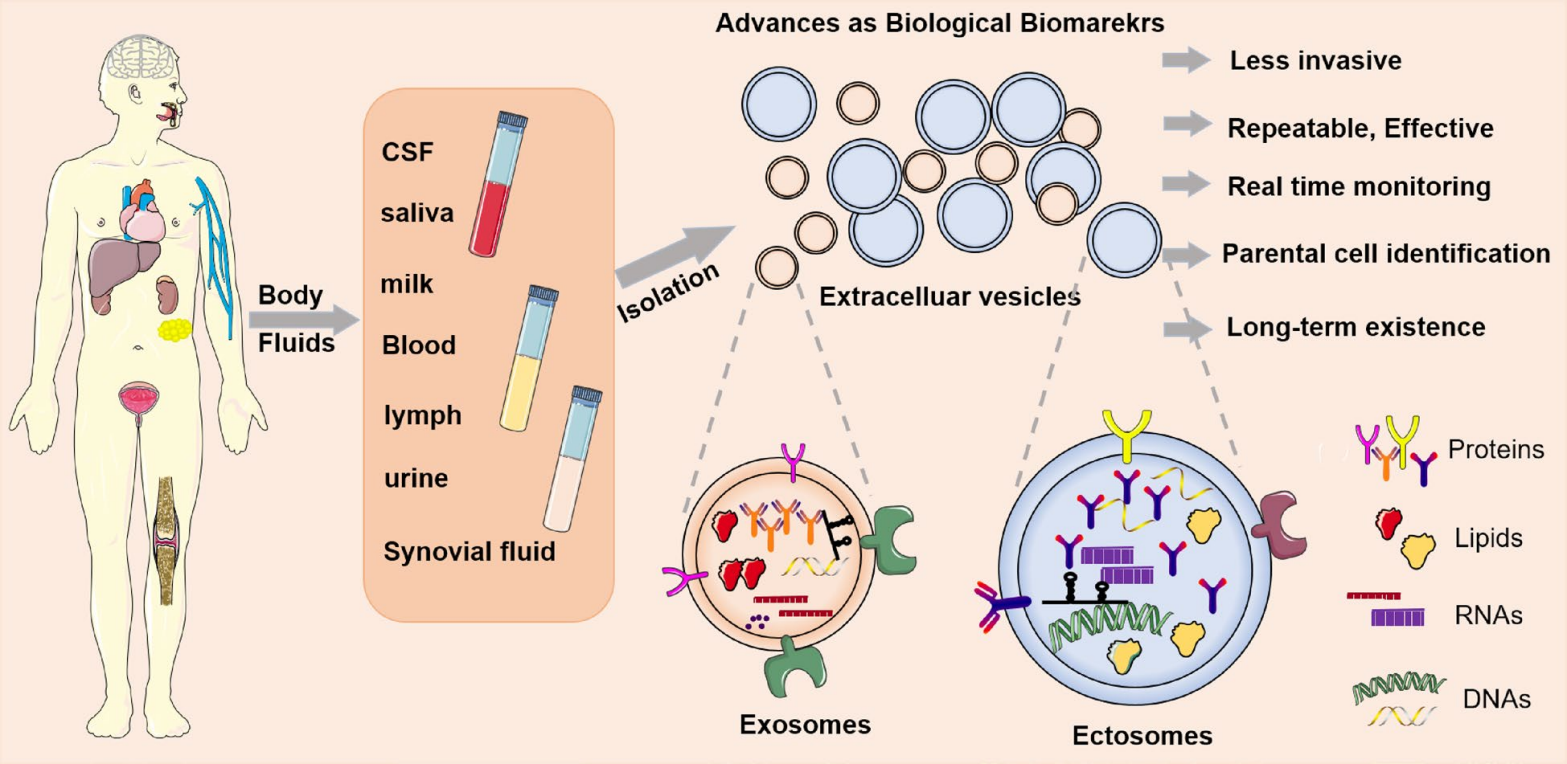
Advances of extracellular vesicles as biological biomarkers. Extracellular vesicles (EVs) can be found in a wide range of body fluids, including cerebrospinal fluid (CSF), saliva, milk, blood, lymph, urine, bile, and synovial fluid. EVs represent a heterogeneous group of cell-derived membranous structures that are categories as exosomes and ectosomes. Exosomes are EVs in the size range of 40–160 nm in diameter, whereas ectosomes are in a size range of 50 nm–1 μm in diameter. EVs have emerged as important modulators in intercellular information transmission by delivering their biological cargo, such as proteins, lipids, RNAs and DNAs. EVs display great superiority in liquid biopsy with the following reasons: (1) the sample collection is less invasive and applicable in different types of diseases; (2) it is time saving, sample repeatable and cost effective; (3) EVs are secreted continuously by living cells, providing exciting opportunities for real time monitoring; (4) the EVs contain differentially expressed cargos and specific surface markers from their parental cells, which could accurately reflect the pathological factors of diseases; (5) the membranous shell of EVs show high stability to guarantee the long-term existence of their contents

Recent evidence identify that the oxidative stress contributes to the release of extracellular vesicles (EVs) [28–30]. EVs are a heterogeneous group of cell-derived membranous structures that are categories as exosomes and ectosomes according to their different mechanism of formation and approximate size [31] (Fig. 1). Exosomes are EVs in the size range of 40–160 nm in diameter with an endosome origin, termed a multivesicular body biogenesis [31, 32]. Different from exosomes, the ectosomes, including microvesicles and microparticles, are ubiquitous vesicles directly released from the plasma membrane through outward budding with a size range of 50 nm–1 μm in diameter [31, 32] (Fig. 1). EVs are derived from almost all types of cells and can be found in diverse biological fluids, including plasma, serum, lymph, saliva, urine, bile, cerebrospinal fluid and human milk [33, 34] (Fig. 2). EVs have emerged as important modulators in intercellular information transmission by delivering their biological cargo, such as proteins, lipids, RNAs and DNAs [35, 36] (Fig. 2).

Currently, emerging evidence have attempted to explain how CS affects the generation and release of EVs, and what is the specific molecular mechanism or pathways they interfered that cause the disease occurrence, especially for lung, cardiovascular and cancer-related diseases. However, little is known about how CS-induced EVs regulate the pathophysiological processes of OLK, even though the oral mucosa is the first-line site affected by CS. Thus, attempts to understand the roles of CS-induced EVs in the microenvironment of OLK are of great importance, which not only helps to clarify the immunopathogenesis of OLK in-depth, but also provides novel strategies for the diagnosis and treatment of OLK, even inhibition of its malignant transformation.

## Advances of EVs as biological biomarkers

Although the traditional tissue biopsies remains the gold standard for disease diagnosis, the procedure is invasive and commonly constrained by its repeatability and brings economic and mental burden to patients [37, 38]. These limitations of traditional tissue biopsy promote the application of biofluids/liquid biopsy technique in clinical practice. Interestingly, EVs display great superiority in liquid biopsy with the following reasons (Fig. 2): first, the prevalence of EVs that exist in almost all body fluids makes it possible to implement less invasive operation and to apply in different types of diseases [33, 34]. Second, it is time saving, sample repeatable and cost effective due to the rapid advancement in EVs-related purification and identification techniques [39, 40]. Third, EVs are secreted continuously by living cells, providing exciting opportunities for real time monitoring of lesions, disease diagnosis, prediction and surveillance [41]. Fourth, the EVs contain differentially expressed cargos and specific surface markers from their parental cells, which could accurately reflect the pathological factors of diseases on both molecular and cellular levels [42, 43]. This might largely contribute to the progression of targeted therapy in future. Finally, the membranous shell of EVs show high stability to guarantee the long-term existence of their contents [44], which would not be affected by the different processing time of samples, ensuring the stable and reliable detection results.

## CS-induced EVs as biomarkers for the diagnosis and treatment of diseases

The exposure of CS could increase the production of EVs from diverse types of cells, including endothelial cells, epithelial cells and fibroblasts, which might rely on the redox modifications of protein thiols [45, 46]. Serban et al. showed that CS-induced endothelial microparticles (EMPs) contained predominantly exosomes that were largely enriched in let-7d, miR-191, miR-126 and miR-125a [47], which may be important in predicting potential function of exosomes as paracrine effectors in patients with chronic obstructive pulmonary disease (COPD). In addition, these EMPs were ceramide-rich and required the ceramide-synthesis enzyme acid sphingomyelinase (aSMase) for microparticle release [47]. The aSMase is an enzyme that is classically associated with stress-induced apoptosis and shown higher activity in plasma of patients with COPD or of CS-exposed mice. These reveal that CS-induced EMPs could be a promising indicator of the functional state of the endothelium in smokers. Stassen et al. identified that CSE promoted the secretion of CD63^+^CD81^+^ extracellular vesicles (exosomes) by bronchial epithelial cells [48]. Moreover, these exosomes were enriched in tissue factor (TF) and exerted a TF-dependent procoagulant activity compared with exosomes by unexposed cells [49], suggesting that TF^+^ exosomes might contribute to the elevated cardiovascular risk in smokers and consider as biomarkers of exposure to CSE.

## The EVs-borne cargo are changed by CS exposure

### The change of proteins in EVs exposure to CS

The protein composition of CS-induced EVs could be influenced by many factors including gender, age, hormones and smoking status. In the case of gender, Varming et al., by analyzing the proteins of plasma exosomes, have found a lower level of amphiregulin (AREG), MUC1, MUC18 (CD146), CD13 and tumor susceptibility gene 101 (TSG101) in female smokers compared to the group of non-smoking females, and this was not seen for male smokers [50]. However, the male smokers showed a higher expression level of CD171, programmed death-ligand 1 (PD-L1) and TSG101 when comparing to the smoking females and non-smoking males [50]. With respect to age, Mobarrez et al. identified higher expression levels of CD41, CD235, TF, and phosphatidylserine (PS) in CS-induced circulating microparticles from younger and older groups compared to those of middle age [51].

In our preliminary study, we have collected the plasma exosomes from patients with OLK and healthy individuals. By proteomic analysis, we identified 10 differentially expressed proteins between the two groups. To be specific, there were 4 upregulated proteins (P01742, Q9BXG8, P06310, P19652) and 6 downregulated proteins (A0A0B4J1X8, Q9NZP8, P61604, Q01459, Q6UXB8, Q9Y5Y7) in patients with CS-induced OLK compared to healthy individuals (data unpublished), suggesting that the alteration of circulating exosomal proteins might play key roles in promoting OLK development and be considered as biomarkers for early diagnosis of OLK.

### The change of cytokines in EVs exposure to CS

CS could change the expression level of cytokine/chemokines in EVs. In patients with CS-induced COPD, the pro-inflammatory Wnt5a and cytokines including interleukin (IL)-8, IL-1β, IL-6 and tumor necrosis factor (TNF)-α in serum EVs were significantly elevated [52]. The functionality of Wnt5a is multifaceted. Apart from as the pro-inflammatory Wnt ligand, it can prohibit the transcription of anti-inflammatory peroxisome proliferator-activated receptor γ and modulate the polarization of M1/M2 macrophages. IL-6, as the NF-κB-dependent cytokines, is a prototypical cytokine and functions like a double-edged sword for the host. The appropriate expression of IL-6 plays a positive role in eliminating infectious agents and restoring damaged tissues, whereas excessive expression of IL-6 exerts a positive role in the progression of different inflammatory diseases even tumors [53]. For patients with OLK, an elevation of salivary IL-6 levels was found compared to healthy controls, with a positive relationship with the severity of dysplasia [54, 55]. Moreover, use of tobacco was seen to significantly promote the elevation of IL-6. These evidence pose the possibility that aberrant expression of EVs-derived IL-6 play a pathological role in the dysplasia of patients with OLK.

Kumar et al. found that the packaging of CD9 in rat plasma-derived EVs was significantly increased after self-administered nicotine, one of the major addictive ingredient in CS [56]. The increased expression of CD9 in plasma EVs might indicate an effect of nicotine on EVs formation, because CD9 is reported an important organizer of proteins and lipid complexes at the EV membrane [57]. In addition, an increased expression of cytochrome P450 (CYP2A6) in plasma EVs was also found [56]. CYP2A6 is responsible for the metabolism of nicotine. These results suggest that the elevated CYP2A6 in plasma EVs upon nicotine self-administered could cause the increased production of toxic metabolites and induction of oxidative stress, and ultimately result in tissue damage and disease progression. The enhanced release of EVs by reactive carbonyl species (RCS) and ROS stimulation could be blocked by antioxidant glutathione *S*-transferase (GSH) [48]. Interestingly, Ye et al. found a significantly accumulation of ROS in CSE-exposed leuk1 cells compared with non-exposed cells [27]. Simultaneously, the Nrf2 was activated in both CS-treated animal model and leuk1 cells, which alleviated smoking-induced oxidative stress [27]. In addition, the use of sulphoraphane (SFN), a natural antioxidant agent present in cruciferous vegetables that activates Nrf2, on leuk1 cells increased the expression of GSH [27]. Thus, these data indicate that SFN might be a promising therapeutic strategy to interfere the release of CS-induced EVs.

### The change of microRNAs (miRNAs) in EVs exposure to CS

The functionality of miRNAs in EVs, especially in exosomes, are of particular interest at present, because the proportion of miRNAs in exosomes accounts for 50%, which is much higher than in their parental cells [58, 59]. Evidence proved that plasma exosomes of different types of smokers including cigarette smokers, waterpipe smokers, electronic cigarettes users and dual smokers have common differential expression of miRNAs, especially hsa-let-7a-5p [60]. Sundar et al. also identified that the hsa-miR-144-3p, hsa-miR-486-5p, hsa-miR-92a-3p and hsa-miR-93-5p were significantly reduced in CS-induced exosomes from plasma compared to non-smokers, whereas the hsa-miR-29a-3p was significantly elevated [61]. Xu et al. found that the level of serum exosomal miR-21 from smokers were higher than those for non-smokers [62]. In addition, the levels of serum exosomal miR-21 inversely correlated with forced expiratory volume in 1 s/forced vital capacity ratio (FEV_1_/FVC%), an indicator representing the percentage of the lung size that can be exhaled in 1 s [62]. Taken together, these findings might pave the way for considering circulating exosomal miRNAs as new biomarkers or theranostic factors in smoking-induced diseases.

It is worth noting that the differentially expressed miRNAs in OLK are identified to associate with its malignant transformation [63–66]. In the latest scoping review, the authors have included fifteen articles for analysis and identified 21 differentially expressed miRNAs in OLK [67]. To be specific, 6 miRNAs (miR-21, miR-181b, miR-184, miR-345, miR-146a and miR-208-3p) that were categories as oncogenic, 5 miRNAs (miR-31*, miR-3065-5p, miR-129-2-3p, miR-204-5p and miR-375) that were classified as tumor suppressors and 10 miRNAs (miR-138, miR-639, miR-27a-3p, miR-15b, miR-485-5p, miR-200c, miR-19a, miR-424, miR-205 and miR-21) that were related to epithelial–mesenchymal transition, invasion and migration [67]. Among these miRNAs, the authors concluded that miR-21, miR-345, miR-181b, miR-31* might be potential markers of malignant transformation of OLK.

### The change of long noncoding RNAs (lncRNAs) in EVs exposure to CS

The lncRNAs are operationally classified as transcripts > 200 nucleotides that do not have any evident protein-coding potential [68, 69]. The distribution of lncRNAs is diverse, which is present in both the nucleus and cytoplasm [70]. LncRNAs are demonstrated to play important roles in multiple biological processed because of their capability to affect gene expression by interacting with DNA, RNAs and proteins [71]. Recently, lncRNAs are found to be selectively packaged into exosomes and function as a messenger in intercellular communication to regulate the recipient cells [72, 73]. Notably, CS exposure could change the lncRNA profile in exosomes. Compared to the non-smokers, Kaur et al. identified 2 upregulated and 5 downregulated lncRNAs in the exosomes from blood plasma of cigarette smokers [74]. In regard to E-cigarette smokers, 13 differentially expressed lncRNAs were found [74]. Among these, the expression level of Bcl2 interacting protein 3-like protein (BNIP3L) revealed approximately fourfold increase [74]. BNIP3L/Nix is a mitochondrial and pro-apoptotic protein integrated in the mitochondrial outer membrane [75]. The most well-accepted mechanism underlying BNIP3L-induced mitophagy is through the binding to Atg8 family including LC3 and BECN1 [76]. Furthermore, BNIP3L-mediated mitophagy might function as a tumor promoter with the evidence that *Bnip3l* depletion significantly delays the progression of pancreatic cancer and improves the survival in a murine model of pancreatic ductal adenocarcinoma [77].

Liu et al. have found that the expression of LC3-II and Beclin-1/BECN1 were upregulated in leuk-1 cells upon CS stimulation and this upregulation was in a concentration- and time-dependent manner [78], suggesting that CS exposure could trigger the autophagy of human oral epithelial cells. Similarly, Lima et al. also confirmed the significant elevation in the percentage of LC3-II positive cells from the normal oral mucosa to the leukoplakia samples, especially in the upper layers of the epithelium [79]. Interestingly, the aberrant expression of lncRNAs was also found in OLK. By performing microarray, Liu et al. for the first time identified a novel key lncRNA n386251 that was highly expressed in OLK. They named it as lncRNA oral leukoplakia progressed associated 1 (LOLA1) because of their ability to affect the migration, invasion, and epithelial–mesenchymal transition of cells, which was associated with the malignant transformation of OLK [80]. In addition to LOLA1, the lncRNA IFITM4P was also significantly upregulated in OLK samples compared to oral normal mucosa and was the highest in OSCC samples [81]. Moreover, IFITM4P could lead to increased cell growth and colony formation in leuk-1 cells [81], indicating the contribution of IFITM4P as an oncogene in oral carcinogenesis.

## CS-induced EVs as mediators for intra/inter-cellular message transmission

The changes of circulating EVs induced by CS are in essence due to the changes of EVs secreted by various cells under the stimulation of CS, and finally released into the peripheral blood or local tissues. The altered composition in EVs upon CS exposure ultimately leads to their dysregulated functions after being engulfed by recipient cells. Researches on the production of EVs, the specific changes of contents, and the mechanism of modulating cells upon CS stimulation could contribute to our deeper understanding in the field of EVs, which will benefits for future use of EVs as diagnostic biomarkers and therapeutic targets of smoke-induced-related diseases.

### The change of epithelial cells-derived EVs exposure to CS

The oral mucosa is the first line that encounters with CS, which makes the epithelial cells in oral cavity are inevitably as the first type of cells affected by CS. However, the researches on oral epithelial cells-derived EVs exposure to CS at present are scare and mainly focused on airway epithelial cells (AECs). The proteomic analysis revealed that about 201 proteins were differentially expressed in BEAS-2B AECs-derived EVs exposed to 1% cigarette smoke condensate (CSE) for 24 h compared with the EVs unexposed to CSE. Among these, 24 proteins, including TF, of the pathway haemostasis were remarkably elevated, indicating the procoagulant properties of CS-induced EVs [49]. In addition, the RNA content of EVs released from human small airway epithelial cells (SAE) were also changed upon exposure to CSE. Corsello et al. proved that a total of 289 miRNAs and 62 piwi-interacting RNA (piRNAs) were changed. To be specific, the expression levels of hsa-miR-3913-5p, hsa-miR-574-5p, hsa-miR-500a-5p and piR-50603 in SAE-derived EVs exposed to CSE were significantly upregulated, whereas the expression levels of hsa-miR-618 and pi-R52900 were significantly downregulated [82]. These data promote to identify biomarkers for diagnosis and novel targets for therapeutic strategies of CS-related diseases.

Notably, the altered composition in EVs implies the alteration of functionality of EVs on the recipient cells. Serban et al. showed that the engulfment of EMPs by peripheral blood monocytes-derived macrophages was correlated with significant inhibition of efferocytosis, indicating that EMPs might play an important role in the pathogenesis of diseases linked to inflammation in response to CS [47]. It was reported that the upregulation of miR-21-3p or miR-27b-3p in CSE-treated Beas-2B-derived exosomes could not only induce macrophages RAW264.7 to express CD86, CD80, CD163 and CD206, but also promote them to produce TNF-α, IL-6, inducible nitric oxide synthase (iNOS), IL-10, Arginase (Arg)-1 and transforming growth factor-beta (TGF-β), suggesting their ability to polarize the macrophages into M1 phenotype and M2 phenotype simultaneously [83]. Interestingly, Zhu et al. found that exposure of CSE could promote the polarization of M2 macrophages (F4/80^+^CD206^+^ cells) with significantly increased expression of *Arg-1* and *IL-10* by investigating the oral mucosa epithelial tissue in OLK mouse model [84]. Taken together, in conjunction with the aforementioned increased expression of exosomal miR-21 in patients with OLK, we propose that exposure of CSE increases the secretion of miR-21^+^ exosomes, which promotes the polarization and expression of *Arg-1* and *IL-10* of M2 macrophages.

Another study reported by Fujita et al. have confirmed that the expression of miR-210 in human bronchial epithelial cells (HBECs)-derived exosomes induced by CS was significantly elevated, which contributed to myofibroblast differentiation by suppressing its target gene *ATG7*, a key composition of autophagy [85]. These results introduced the involvement of autophagy processes mediated by CS-induced exosomes that might open a novel path for us to better understand the pathological process of diseases [85]. Evidence showed that, under the CS stimulation, the autophagy of oral epithelial cells also changed. To be specific, the classical autophagy-related proteins LC3-II and Beclin-1 were upregulated in a concentration- and time-dependent manner [78]. As we mentioned above, the LC3-II was also overexpressed in the leukoplakia samples, especially in the upper layers of the epithelium, compared to the normal tissues [79]. Based on the data, we infer that the alteration of CSE-induced EVs might upregulate the autophagy of oral mucosal epithelial cells by upregulating LC3-II and Beclin-1, and ultimately contributes to the progression of OLK.

It has been found that the CS exposure of bronchial epithelial cells enhanced the encapsulation of matured miR-93 in EVs. These EVs-born miR-93 that were taken up by macrophages suppressed dual-specificity phosphatase 2 (DUSP2) and then activated the JNK pathway, which upregulated the expression of Matrix metalloproteinases (MMP)-9 and MMP-12 and leaded to elastin degradation [86]. The MMP-9 is one of the most complex MMPs and belongs to gelatinase family to degrade the extracellular matrix (ECM) components during tissue remodeling, which plays an essential role in tumor invasion and metastasis [87, 88]. The expression of MMP-9 has proven to be a reliable diagnostic and prognostic marker in oral cancer [89–91]. Prior studies also have documented the significant elevation of MMP-9 in patients with OLK [92–94]. In addition, the expression of MMP-9 increased higher in lesions with more severe dysplasia compared to those with lower degree of dysplasia, indicating their positive correlation with the disease progression.

### The change of macrophages-derived EVs exposure to CS

CS was proven to exert different effects on the release of EVs from different types of macrophages. Specifically, exposure to CS contributes to the production of EVs from proinflammatory macrophages, but inhibits the secretion of EVs from anti-inflammatory macrophages. For example, the expression level of MMP-14 in EVs from proinflammatory macrophages are 3 times high after exposed to CS than non-exposed controls [95]. This increase could greatly affect the tissue remodeling and fibrogenesis due to the gelatinolytic and collagenolytic activity of MMP-14. Among the MMPs family, MMP-14 shows the broadest substrate specificity of all membrane-bound MMPs, especially to the composition of pericellular ECMs, which affects the cell–cell or cell–ECM communication [96, 97]. The effects are versatile including the promotion of matrix degradation and remodeling, angiogenesis, inflammation, tumor invasion and metastasis [96, 97]. Thus, the enrichment of MMP-14^+^ MVs might be a mediator that destroy tissue integrity and organs of individuals exposed to CS. It is reported that the suppressor of cytokine signaling (SOCS) 1 and SOCS3 in EVs taken up by epithelial cells downregulate the inflammatory cytokine signaling under homeostatic conditions [98]. However, after CS exposure, both SOCS1 and SOCS3 in EVs from anti-inflammatory macrophages are decreased, indicating the aggravation of inflammation [98].

### The change of T cells-derived EVs exposure to CS

Apart from macrophages, it was reported that exposure of CS can also change the component of T cells-derived EVs and arouse the dysregulation of EVs functions. Donate et al. found that the Aryl hydrocarbon receptor (AhR) on Th17 cells were activated upon CS exposure, and then resulted in the increased expression of EVs-loaded miR-132 derived from Th17 cells. These CS-induced EVs had no obvious effect on the number and cytokine production (TNF, IL-6, IL-23) of dendritic cells, as well as the polarization of M0 macrophages, but they acted as a proinflammatory factor that induced osteoclastogenesis by inhibiting cyclooxygenase-2 (COX-2), revealing a mechanism of CS signaling and offering a potential target for therapeutic interference of inflammatory disease [99].

### The change of monocytes-derived EVs exposure to CS

Interestingly, the human mononuclear cells-derived EVs stimulated by CSE also manifested a proinflammatory effect. Cordazzo et al. found a rapid generation of MPs by mononuclear cells upon exposure to CSE for 15 min on calcium-dependent mechanism. The MPs from CSE treated mononuclear cells showed a 2.6-fold, significant increase of TF activity indicating their elevated procoagulant potential [100]. Moreover, these MPs increased the expression of proinflammatory mediators including IL-18, intercellular adhesion molecule (ICAM-1), and monocyte chemoattractant protein (MCP)-1 of lung epithelial cells [100]. These data well-supplemented Li’ observations who identified that the enhanced production of MPs was induced by apoptosis of human monocytes/macrophages exposed to CSE [101].

## Conclusions

**Fig. 3:**
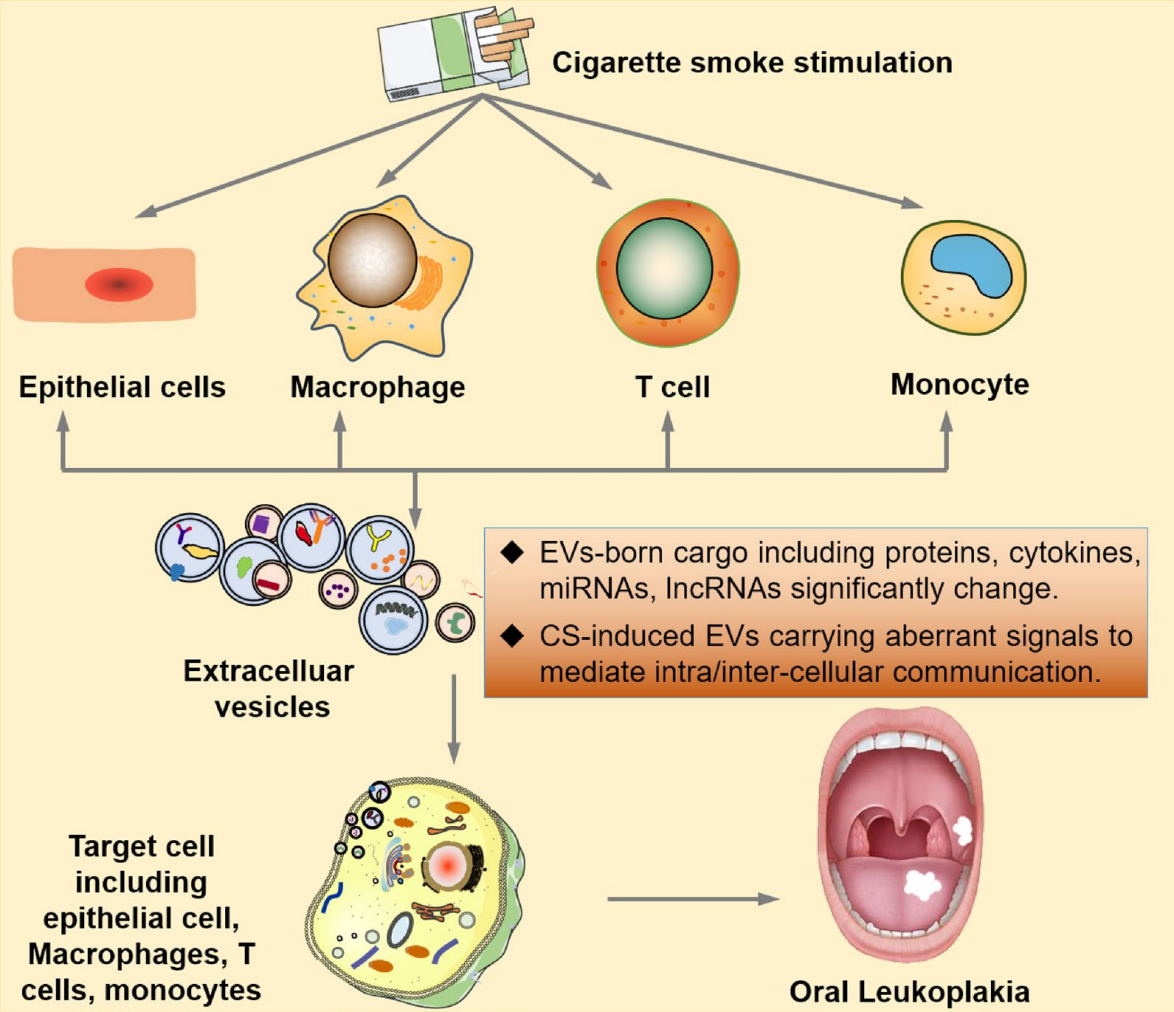
Potential roles of CS-induced EVs in promoting oral leukoplakia. Exposure to cigarette smoke contributes to the production of EVs from different types of cells including epithelial cells, macrophages, T cells, and monocytes. The cargo of these EVs induced by CS stimulation are significantly changed. These bioactive cargo contain proteins, cytokines, RNAs, and DNAs, which exerts mediators for intra/inter-cellular communication. When these EVs are engulfed, they can change the biological behavior of the recipient/target cells and lead to the onset of pathogenesis. The series of changes ultimately might result in the initiation of oral leukoplakia or even carcinogenesis. *EVs* extracellular vesicles, *CS* cigarette smoke

The health of oral mucosal epithelial is prone to be damaged, because it is the first affected site by CS. Exposure to CS is identified as a major risk factor for OLK and its malignant transformation to OSCC. However, evidence done on the role of EVs in CS-induced OLK is still in its infancy. Based on the prior literature, upon CS stimulation, the biogenesis of EVs is significantly elevated, the composition of EVs and the signaling transduction via EVs are obviously changed, which is closely associated with the occurrence and progression of disease. Exposure to cigarette smoke contributes to the production of EVs from different types of cells including epithelial cells, macrophages, T cells, and monocytes. The bioactive cargo (proteins, cytokines, RNAs, and DNAs) of these EVs induced by CS stimulation are significantly changed, which acts as mediators for intra/inter-cellular communication. When these EVs are engulfed, they can change the biological behavior of the recipient/target cells and finally lead to the onset of OLK pathogenesis or even carcinogenesis (Fig. 3). Further researches should be performed to identify these assumptions. Elucidating the functions of CS-induced EVs in the pathophysiological processes of OLK is of great significance for the development of diagnostic biomarkers, prognostic evaluation, and therapeutic targets of OLK.

## Data Availability

Not applicable.
